# Cutaneous small vessel vasculitis following calcium-hydroxyapatite filler: A rare case report

**DOI:** 10.1177/2050313X251341563

**Published:** 2025-06-13

**Authors:** Ghassan Barnawi, Sarah Kashkari, Arieh Gomolin, May Chergui, Badia Issa-Chergui, Lisa Iannatone

**Affiliations:** 1 Department of Dermatology, McGill University, Montreal, QC, Canada; 2 Department of Pathology, McGill University, Montreal, QC, Canada

**Keywords:** vasculitis, filler, cutaneous, small

## Abstract

This case report describes a 61-year-old female who developed cutaneous small vessel vasculitis following a calcium-hydroxyapatite-based filler injection. The patient presented with a generalized purpuric and petechial rash, along with arthralgia. A skin biopsy confirmed features consistent with cutaneous small vessel vasculitis. Treatment with a 6-week prednisone taper resulted in complete symptom resolution. This observation warrants further investigation into the potential role of dermal fillers in triggering inflammatory conditions like cutaneous small vessel vasculitis.

## Case presentation

A 61-year-old female presented with a 3-day history of a widespread rash characterized by purpuric and petechial lesions, beginning on the lower extremities and spreading to the torso and arms. Accompanying arthralgia in the small joints of the hands and feet was noted, without systemic or constitutional symptoms. She had not taken any new medications in the preceding 14 days; however, the day before the onset of the eruption, she had received a calcium-hydroxyapatite (CaHA)-based filler treatment by a licensed professional.

Physical examination revealed nonblanchable petechiae and palpable purpura involving the lower and upper extremities as well as the trunk. A biopsy revealed small vessel vasculitis, and direct immunofluorescence was negative. Autoimmune and inflammatory markers were unremarkable. Treatment involved a prednisone taper for 6 weeks, resulting in complete resolution of the eruption and accompanying arthralgia.

## Discussion

Cutaneous small vessel vasculitis (CSVV) is an inflammatory condition affecting the small blood vessel walls in the superficial and mid dermis, often without systemic involvement.^1^ In most cases, CSVV is idiopathic, though it can be triggered by medications, infections, or hematologic conditions.^2,3^ CaHA fillers are widely used for soft tissue augmentation and are generally considered safe.^4^ Adverse effects are typically mild and localized, such as erythema, swelling, or nodules from overfilling.^4^ Severe complications, such as vascular occlusion or granuloma formation, are rare, and systemic reactions have not been previously reported in association with CaHA fillers.^4^ In this report, CSVV was observed shortly after CaHA filler injection. While the exact mechanism remains unclear, this observation suggests the possibility of the filler acting as a precipitant in a predisposed individual. Further research is needed to determine whether there is an association and to explore underlying immunologic mechanisms

## Conclusion

**Figure 1.**
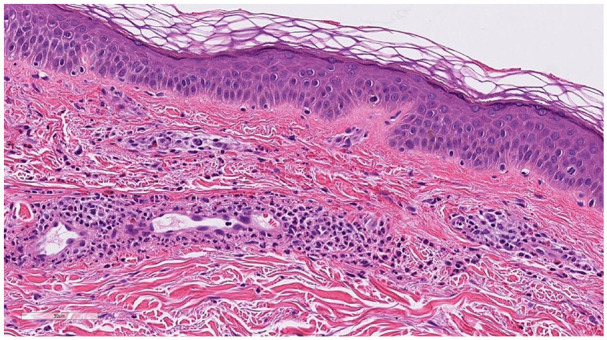
H&E stain at high magnification shows small vessel vasculitis characterized by a perivascular neutrophilic infiltrate with mild eosinophils and red blood cell exocytosis. H&E: hematoxylin and eosin.

This case highlights the rare observation of CSVV following CaHA filler injection. While CSVV is typically triggered by infections, medications, or hematologic conditions, the potential role of dermal fillers warrants further investigation to confirm any association and understand the underlying mechanisms (Figure 1).
